# Digital healthy eating literacy: its role in sustainable food choices and mediterranean diet adherence

**DOI:** 10.1186/s12889-025-23353-4

**Published:** 2025-06-05

**Authors:** Busra Aslan Gonul, Zeynep Caferoglu Akin

**Affiliations:** 1 Department of Nutrition and Dietetics, Faculty of Health Sciences, Erciyes University, Kayseri, 38260 Türkiye; 2 Department of Health Sciences, University of York, York, UK

**Keywords:** Digital nutrition literacy, Mediterranean diet, Sustainable diets, Environmentally responsible food choices, Health literacy, Planetary health

## Abstract

**Background:**

The rise of digital platforms as sources of nutrition information has highlighted the need for digital healthy eating literacy to ensure informed dietary choices. Understanding the role of digital healthy eating literacy in shaping sustainable dietary behaviours is crucial for promoting both individual and planetary health. This study aimed to examine the associations between digital healthy eating literacy, environmentally responsible food choices, and adherence to the Mediterranean diet (MedDiet), a sustainable dietary model, among adults in Türkiye.

**Methods:**

A total of 1,516 adults (mean age: 28.9 ± 9.8 years) residing in Türkiye were recruited through an online survey distributed via social platforms. Participants completed an online questionnaire comprising the e-Healthy Diet Literacy (e-HDL) Questionnaire, the Environmentally Responsible Food Choice Scale, and the 14-item Mediterranean Diet Adherence Screener (MEDAS). This cross-sectional study employed multivariable regression analyses to examine the associations between digital healthy eating literacy, environmentally responsible food choices, and adherence to the MedDiet.

**Results:**

Higher e-HDL scores were significantly associated with increased environmentally responsible food choices (β = 0.283, 95% CI: 0.233–0.333, *p* < 0.001) and a 1.039-fold increase in the odds of adherence to the MedDiet (95% CI: 1.021–1.058, *p* < 0.001). The strength of the association between e-HDL and the outcomes varied by subgroup, with stronger associations observed for environmentally responsible food choices among non-smokers, non-drinkers, daily exercisers, and higher-income individuals, and for adherence to the MedDiet among women, non-smokers, non-drinkers, and those with lower or equal income levels. Among the MedDiet adherents, e-HDL explained 10.5% of the variance in environmentally responsible food choices (*p* < 0.001).

**Conclusions:**

This study highlights the potential importance of digital healthy eating literacy in relation to sustainable dietary behaviours and environmental health. Targeted digital nutrition education programmes may help support improvements in digital healthy eating literacy and encourage sustainable diets, supporting planetary health. Future policies should focus on increasing awareness and accessibility of reliable nutrition information on digital platforms to improve dietary practices and environmental sustainability.

**Supplementary Information:**

The online version contains supplementary material available at 10.1186/s12889-025-23353-4.

## Introduction

Environmental issues such as global warming and air and water pollution are becoming increasingly significant global challenges [1]. Food production and supply chains contribute to 20–30% of humanity’s total environmental impact due to factors such as resource depletion, environmental pollution, biodiversity loss, greenhouse gas emissions and rising global temperatures [1, 2]. Meat production, in particular, is a major source of greenhouse gas emissions and causes environmental problems such as terrestrial acidification and eutrophication due to the intensive use of land and freshwater resources [2]. While meat substitutes have recently gained attention as alternatives to reduce the environmental burden of animal-based diets, ultra-processed plant-based meat alternatives may also pose challenges to environmental sustainability due to resource-intensive production processes [3]. Processed foods high in sugar, salt, and saturated fats further harm biodiversity and threaten the ecosystem of food chains. In contrast, grains, vegetables, and other plant-based foods have a substantially lower ecological footprint [4, 5]. Adopting ecological eating behaviours, such as choosing fresh and environmentally friendly foods, purchasing local products, and reducing meat consumption, is crucial for environmental sustainability [4].

In addition to environmentally conscious food choices, adherence to sustainable diets is essential for protecting environmental health [6]. Among the recognised sustainable diets, the Mediterranean diet (MedDiet) stands out. This diet is characterised by high consumption of fruits, vegetables, whole grains, legumes, nuts and olive oil; moderate consumption of fish, seafood, eggs, dairy products and poultry; and low consumption of red and processed meats, as well as discretionary foods high in added sugars, salt, and unhealthy fats. As a predominantly plant-based diet, the MedDiet contributes significantly to planetary health by improving soil, water, and air quality due to its low ecological footprint [7]. Compared to animal-based diets, it promotes sustainable resource management by reducing energy consumption and the use of natural resources, making it an environmentally friendly dietary model [7, 8]. Additionally, the MedDiet encourages frugality and conviviality, which are also important for environmental sustainability. Adherence to the MedDiet has been shown to increase sustainable eating behaviours, reduce ecological footprints and lower greenhouse gas emissions [7, 9]. Therefore, adherence to sustainable diets such as the MedDiet is a critical area for improving environmental sustainability.

Nutrition literacy is a key factor in helping individuals make informed and healthy food choices to promote health and protect the environment [6]. Defined as the ability to acquire, process, and understand nutrition-related information and skills, nutrition literacy influences food and dietary choices [10, 11]. With the increasing prevalence of digitalisation, individuals are exposed to a wide range of information through social media platforms. Digital nutrition platforms, such as mobile applications (e.g., MyFitnessPal, Yuka) and social media-based health communities, have become powerful tools due to their ability to reach large audiences, cost-effectiveness, and audience engagement [12]. Research has shown that digital nutrition education has a greater impact on nutrition literacy compared to traditional methods such as brochures and computer games [13]. However, in the presence of inconsistent and contradictory information sources, digital healthy eating literacy has emerged as a critical area for distinguishing accurate, impartial, and reliable nutritional information [12].

While nutrition literacy is known to be associated with both adherence to the MedDiet and food preferences [6, 10, 11], the role of digital healthy eating literacy in relation to these behaviours has not yet been explored. Therefore, this study aims to examine the association between individuals’ digital healthy eating literacy, environmentally responsible food choices, and adherence to the MedDiet, a sustainable dietary pattern.

## Materials and methods

### Participants and procedure

This cross-sectional and descriptive study included 1,643 adults aged 18–65 years residing in Türkiye who reported following at least one social media account related to healthy eating and consented to participate. Recruitment took place over six months (November 2023 to April 2024) via voluntary response and snowball sampling through social media platforms and messaging apps (e.g., Instagram, WhatsApp). The survey link was distributed via public posts and direct messages, inviting eligible individuals to participate.

To confirm eligibility, participants were asked a screening question: “Do you follow any social media accounts that share posts about healthy eating?” Only those who responded “yes” were allowed to proceed. No restrictions were placed on the type of account followed; these could include content shared by health professionals, public organisations, or independent creators using formats such as visuals, text, or video.

Participants were encouraged to share the survey within their networks to increase reach. Exclusion criteria included being a student or graduate of a nutrition and dietetics programme, having a physical or mental disability, or being pregnant or breastfeeding—conditions that could influence dietary behaviours and nutritional needs. After excluding 127 participants who did not meet the inclusion criteria, 1,516 participants from all geographic regions of Türkiye were included in the analysis.

Ethical approval was obtained from the Social and Humanities Ethics Committee of Erciyes University (Date: 31 October 2023; Approval Number: 2023/443). The study adhered to the Declaration of Helsinki. Participants were informed about the study and gave electronic informed consent before completing the online questionnaire. Those who did not consent were unable to access the survey.

### Measures

Data were collected using an online survey on Google Forms, which included the following instruments: a personal information form (including general and health information, as well as internet usage habits), the E-Healthy Diet Literacy (e-HDL) Questionnaire, the Environmentally Responsible Food Choice Scale, and the Mediterranean Diet Adherence Screener (MEDAS). Anthropometric measurements, including weight and height, were self-reported by participants and classified according to World Health Organisation standards [14].

## E-Healthy diet literacy questionnaire

The e-HDL questionnaire, developed by Duong et al. [12] and validated for the Turkish population by Karahan Yilmaz et al. [15], was used to measure participants’ ability to access, understand, evaluate, and apply internet-based healthy diet information. It consists of 11 items distributed across four subdimensions:

- ***Finding.*** Measures the frequency of searching for healthy diet information through institutional or non-institutional channels on a scale from 1 (never) to 5 (daily).
- ***Understanding.*** Assesses the accuracy of comprehension regarding specific nutrition-related statements often found online, with correct answers scored as 5 and incorrect answers as 1.
- ***Judging.*** Evaluates participants’ perceived trustworthiness of various online sources of healthy diet information on a scale from 1 (strongly disagree) to 5 (strongly agree).
- ***Applying***. Measures the frequency of actions taken based on healthy diet information, such as consulting healthcare professionals, on a scale from 1 (never) to 5 (all the time).

In the *finding* and *applying* domains, participants were asked how often they search for healthy nutrition information or respond to misleading online content, and whether they consult healthcare professionals about online health information. The *understanding* domain focused on participants’ ability to comprehend online nutrition content and identify misleading or promotional information. The *judging* domain assessed how participants evaluate the credibility of various digital information sources, reflecting their skills in verifying the reliability of online content.

The total score is calculated by summing the scores of all items, with higher scores indicating greater digital healthy eating literacy. The maximum possible score is 55, indicating the highest attainable level of digital healthy eating literacy. The full list of items and scoring details is provided in Supplementary Table 1.

## Environmentally responsible food choice scale

Participants’ environmentally responsible food preferences were assessed using the Environmentally Responsible Food Choice Scale, developed by Başar et al. [16]. The seven items include:

- “I can pay more for organically grown food”.
- “I avoid consuming food with genetically modified organisms (GMOs)”.
- “I prefer consuming eco-labelled food”.
- “I am careful not to consume too much meat”.
- “I prefer buying dairy products from local producers”.
- “I avoid consuming imported food, such as exotic fruits”.
- “I avoid consuming canned, ready-made food”.

This seven-item scale uses a five-point Likert scale, ranging from 1 (strongly disagree) to 5 (strongly agree). Higher scores indicate a stronger preference for environmentally responsible food consumption. The maximum total score is 35. The full item list and scoring information are presented in Supplementary Table 2.

## Mediterranean diet adherence screener

The MEDAS, developed by Martínez-González et al. [17] and validated for the Turkish population by Pehlivanoğlu et al. [18], was used to evaluate adherence to the Mediterranean dietary pattern. The scale comprises 14 items assessing dietary practices such as using olive oil as the primary fat, consuming fruits, vegetables, legumes, fish, and nuts, and limiting red meat, sugar-sweetened beverages, and commercial pastries.

Each item is scored as 0 or 1, with total scores ranging from 0 to 14. In accordance with the Turkish validation study of the scale [18], total scores of 7 or more indicate acceptable adherence to the MedDiet, while scores of 9 or more reflect strict adherence. The details of the items and scoring method for the MEDAS are presented in Supplementary Table 3.

### Statistical analyses

#### Sample size

According to data from the Turkish Statistical Institute, the population aged 18–65 in Türkiye is approximately 54,249,506 [19]. Based on a 95% confidence level and a 5% margin of error, the required sample size was calculated as 385 using a simple random sampling method (http://www.raosoft.com). Considering an expected response rate of 25%, the target sample size was increased to at least 1,540 participants. Although following a social media account related to healthy eating was an inclusion criterion, the sample size was calculated based on the general adult population, as no reliable national data exist on the size of this specific subgroup.

### Data analysis

All statistical analyses were conducted using IBM SPSS Statistics, version 27.0. Categorical variables were summarised as numbers (n) and percentages (%) and compared using chi-square tests. The normality of continuous variables (age, BMI, and scale scores) was assessed through visual inspection (histograms and Q–Q plots) and the Kolmogorov–Smirnov test. Since all continuous variables were normally distributed, they were presented as mean ± standard deviation (SD), and group differences were analysed using Student’s t-test.

Multivariable linear regression analyses were performed to evaluate the associations between e-HDL scores (independent variable) and environmentally responsible food choices (dependent variable). Regression coefficients (β) and 95% confidence intervals (CI) were reported. The initial model was adjusted for age, gender, and body mass index (BMI). In the subsequent model, additional sociodemographic variables—marital status, educational status, working status, and nutrition education—were included. To explore whether the associations varied across population subgroups, the analyses were stratified by smoking status, alcohol consumption, exercise within the last 30 days, and income level.

Multivariable logistic regression analyses were conducted to examine the association between e-HDL scores and adherence to the MedDiet. For this analysis, adherence was dichotomised as nonadherent (< 7) and adherent (≥ 7) based on scores from the 14-item MEDAS screener. Odds ratios (OR) and 95% confidence intervals (CI) were reported. The initial model was adjusted for age, BMI, and exercise. The subsequent model included the same four sociodemographic variables as in the linear regression analyses. Stratified analyses were also conducted by gender, smoking status, alcohol consumption, and income level.

Both regression analyses used a backward stepwise technique. All potential explanatory variables were initially entered into the model. Variables with a significant Wald test at a level of 0.25 were retained in the multivariate analysis, while non-significant variables were removed stepwise. Model fit was assessed by comparing adjusted R² (linear regression) and Nagelkerke R² values (logistic regression). Potential multicollinearity was checked, and no violations were observed.

In addition, the linear regression model was stratified by the MedDiet adherence (nonadherent vs. adherent) to explore potential differences in the association between e-HDL scores and environmentally responsible food choices. Quadratic regression models were used to determine the best-fitting curve for these stratified associations. For all analyses, statistical significance was defined as *p* < 0.05.

## Results

This study included 1,516 adult participants (mean age: 28.9 ± 9.8 years). The majority were women (70%), had a normal BMI (57.3%), were single (62.9%), held a university degree or higher (73.4%), were employed (56.7%), had an income equal to expenses (55.8%), were non-smokers (69.1%), and non-drinkers (84.8%), and had not received any formal nutrition education (83.4%). Descriptive statistics and scale scores across the MedDiet adherence groups are presented in Table 1.

**Table 1:** Descriptive participant characteristics in the entire sample and in groups based on their adherence to the Mediterranean diet

Variables^a^	All participants (*n* = 1516)		Adherence to the MedDiet (cut-off: <7 vs. ≥ 7)				Chi^2^/t	*p*
			Nonadherent (*n* = 910)		Adherent (*n* = 606)			
	*n*	%	*n*	%	*n*	%		
Age (years), x̄±SD	28.9 ± 9.8		28.4 ± 9.7		29.6 ± 10.1		-2.367	0.018^*^
<25	729	48	469	52	260	43	11.060	0.004^**^
25–39	528	35	293	32	235	39		
≥40	259	17	148	16	111	18		
Gender								
Male	455	30	264	29	191	32	1.089	0.297^**^
Female	1061	70	646	71	415	69		
BMI (kg/m^2^), x̄±SD	24.3 ± 4.2		24.3 ± 4.1		24.3 ± 4.3		-0.174	0.862^*^
Underweight (< 18.5)	71	5	43	5	28	5	0.237	0.971^**^
Normal weight (18.5–24.9)	869	57	525	58	344	57		
Overweight (25–29.9)	439	29	262	29	177	29		
Obese (≥ 30)	137	9	80	9	57	9		
Marital status								
Never married	953	63	599	66	354	58	8.552	0.003^**^
Ever married	563	37	311	34	252	42		
Educational status								
High school and below	403	27	236	26	167	28	0.491	0.483^**^
University and over	1113	73	674	74	439	72		
Working status								
Not working	624	41	413	45	211	35	19.919	< 0.001^**^
Working	860	57	484	53	376	62		
Retired	32	2	13	1	19	3		
Income status								
Less than expenses	400	26	243	27	157	26	3.759	0.153^**^
Equal to expenses	846	56	519	57	327	54		
More than expenses	270	18	148	16	122	20		
Smoking (yes)	469	31	271	30	198	33	1.425	0.233^**^
Alcohol consumption (yes)	231	15	136	15	95	16	0.151	0.698^**^
Exercise last 30 days								
Not at all	625	41	423	47	202	33	30.936	< 0.001^**^
Few times a month	378	25	219	24	159	26		
Few times a week	423	28	227	25	196	32		
Everyday	90	6	41	5	49	8		
Nutrition Education								
Took a few courses on nutrition at school	212	14	126	14	86	14	0.058	0.971^**^
Received special nutrition training	39	3	23	2	16	3		
Untrained	1265	83	761	84	504	83		
e-HDL score (max score 55), x̄±SD	27.2 ± 6.1		26.6 ± 5.8		28.1 ± 6.3		-4.569	< 0.001^*^
Environmentally responsible food choices score (max score 35), x̄±SD	22.7 ± 6.2		22.5 ± 5.8		22.9 ± 6.9		-0.959	0.338^*^
Adherence to the MedDiet scores, x̄±SD	6.0 ± 1.9		4.7 ± 1.1		7.9 ± 1.0		-57.151	< 0.001^*^

Abbreviations: BMI, body mass index; e-HDL, e-Healthy Diet Literacy; MedDiet, Mediterranean Diet

^*^Student’s t-test; ^**^Chi^2^-test

^a^Values were given as the number (n) and percentage (%) for qualitative variables and mean (x̄) and standard deviation (SD) for quantitative variables

As shown in Table 2, higher e-HDL scores were associated with more environmentally responsible food choices (Model 2: β = 0.283, 95% CI: 0.233, 0.333; *p* < 0.001). This association was stronger among non-smokers, non-drinkers, individuals who exercised daily, and those with higher income levels (*p* < 0.001).

**Table 2:** Multivariable linear regression analysis for the associations between e-healthy diet literacy and environmentally responsible food choices

	Unadjusted Model			Adjusted Model 1^a^			Adjusted Model 2^b^		
	β	95% CI	*p*	β	95% CI	*p*	β	95% CI	*p*
All participants	0.285	0.235, 0.335	< 0.001^*^	0.295	0.245, 0.345	< 0.001^*^	**0.283**	**0.233**,** 0.333**	**< 0.001** ^*^
Smoking									
Yes	0.242	0.153, 0.330	< 0.001^*^	0.256	0.167, 0.345	< 0.001^*^	0.253	0.163, 0.343	< 0.001^*^
No	0.307	0.247, 0.367	< 0.001^*^	0.314	0.254, 0.374	< 0.001^*^	**0.299**	**0.238**,** 0.360**	**< 0.001** ^*^
Alcohol consumption									
Yes	0.144	0.024, 0.263	0.019^*^	0.145	0.025, 0.266	0.019	0.134	0.013, 0.255	0.030
No	0.316	0.261, 0.370	< 0.001^*^	0.327	0.272, 0.381	< 0.001^*^	**0.315**	**0.260**,** 0.370**	**< 0.001** ^*^
Exercise last 30 days									
Few times a week or less	0.265	0.214, 0.316	< 0.001^*^	0.276	0.225, 0.327	< 0.001^*^	0.265	0.213, 0.316	< 0.001^*^
Everyday	0.545	0.323, 0.767	< 0.001^*^	0.535	0.311, 0.759	< 0.001^*^	**0.539**	**0.306**,** 0.772**	**< 0.001** ^*^
Income rate^c^									
Income ≤ expense	0.252	0.197, 0.307	< 0.001^*^	0.265	0.210, 0.320	< 0.001^*^	0.250	0.195, 0.306	< 0.001^*^
Income > expense	0.413	0.297, 0.529	< 0.001^*^	0.389	0.272, 0.505	< 0.001^*^	**0.391**	**0.272**,** 0.511**	**< 0.001** ^*^

^*^p-trend < 0.05

^a^Model 1 was adjusted for age, gender, and body mass index

^b^Model 2 was further adjusted for marital status, educational status, working status, and nutrition education, in addition to the variables included in Model 1

^c^Income rate categories were based on participants’ self-perceptions: “Income ≤ expense” indicates income less than or equal to expenses; “Income > expense” indicates income more than expenses

In the logistic regression analysis (Table 3), higher e-HDL scores were associated with a 1.039-fold increase in the odds of adherence to the MedDiet (95% CI: 1.021, 1.058; *p* < 0.001), meaning that individuals with higher levels of digital healthy eating literacy tended to show greater adherence to the MedDiet. The strength of this association was greater among women (OR = 1.048, 95% CI: 1.025, 1.073; *p* < 0.001), non-smokers (OR = 1.048, 95% CI: 1.025, 1.071; *p* < 0.001), non-drinkers (OR = 1.042, 95% CI: 1.021, 1.062; *p* < 0.001), and those with lower or equal income levels (OR = 1.045, 95% CI: 1.024, 1.066; *p* < 0.001).

**Table 3:** Multivariable logistic regression analysis for the effect of e-healthy diet literacy on adherence to the Mediterranean diet

	Unadjusted Model			Adjusted Model 1^a^			Adjusted Model 2^b^		
	OR	95% CI	*p*	OR	95% CI	*p*	OR	95% CI	*p*
All participants	1.041	1.023, 1.059	< 0.001^*^	1.039	1.021, 1.057	< 0.001^*^	**1.039**	**1.021**,** 1.058**	**< 0.001** ^*^
Gender									
Male	1.029	1.000, 1.058	0.052	1.022	0.993, 1.053	0.137	1.023	0.993, 1.054	0.128^*^
Female	1.048	1.026, 1.071	< 0.001^*^	1.049	1.026, 1.073	< 0.001^*^	**1.048**	**1.025**,** 1.073**	**< 0.001** ^*^
Smoking									
Yes	1.029	1.000, 1.060	0.051	1.026	0.996, 1.057	0.095^*^	1.027	0.996, 1.060	0.088^*^
No	1.048	1.026, 1.071	< 0.001^*^	1.047	1.024, 1.070	< 0.001^*^	**1.048**	**1.025**,** 1.071**	**< 0.001** ^*^
Alcohol consumption									
Yes	1.033	0.992, 1.076	0.116	1.028	0.985, 1.072	0.201	1.030	0.987, 1.075	0.179
No	1.043	1.023, 1.063	< 0.001^*^	1.041	1.021, 1.061	< 0.001^*^	**1.042**	**1.021**,** 1.062**	**< 0.001** ^*^
Income rate^c^									
Income ≤ expense	1.045	1.025, 1.066	< 0.001^*^	1.043	1.023, 1.064	< 0.001^*^	**1.045**	**1.024**,** 1.066**	**< 0.001** ^*^
Income > expense	1.022	0.985, 1.062	0.248	1.018	0.979, 1.058	0.371	1.014	0.975, 1.055	0.489

^*^p-trend < 0.05

^a^Model 1 was adjusted for age, body mass index, and exercise

^b^Model 2 was further adjusted for marital status, educational status, working status, and nutrition education, in addition to the variables included in Model 1

^c^Income rate categories were based on participants’ self-perceptions: “Income ≤ expense” indicates income less than or equal to expenses; “Income > expense” indicates income more than expenses

**Fig. 1:**
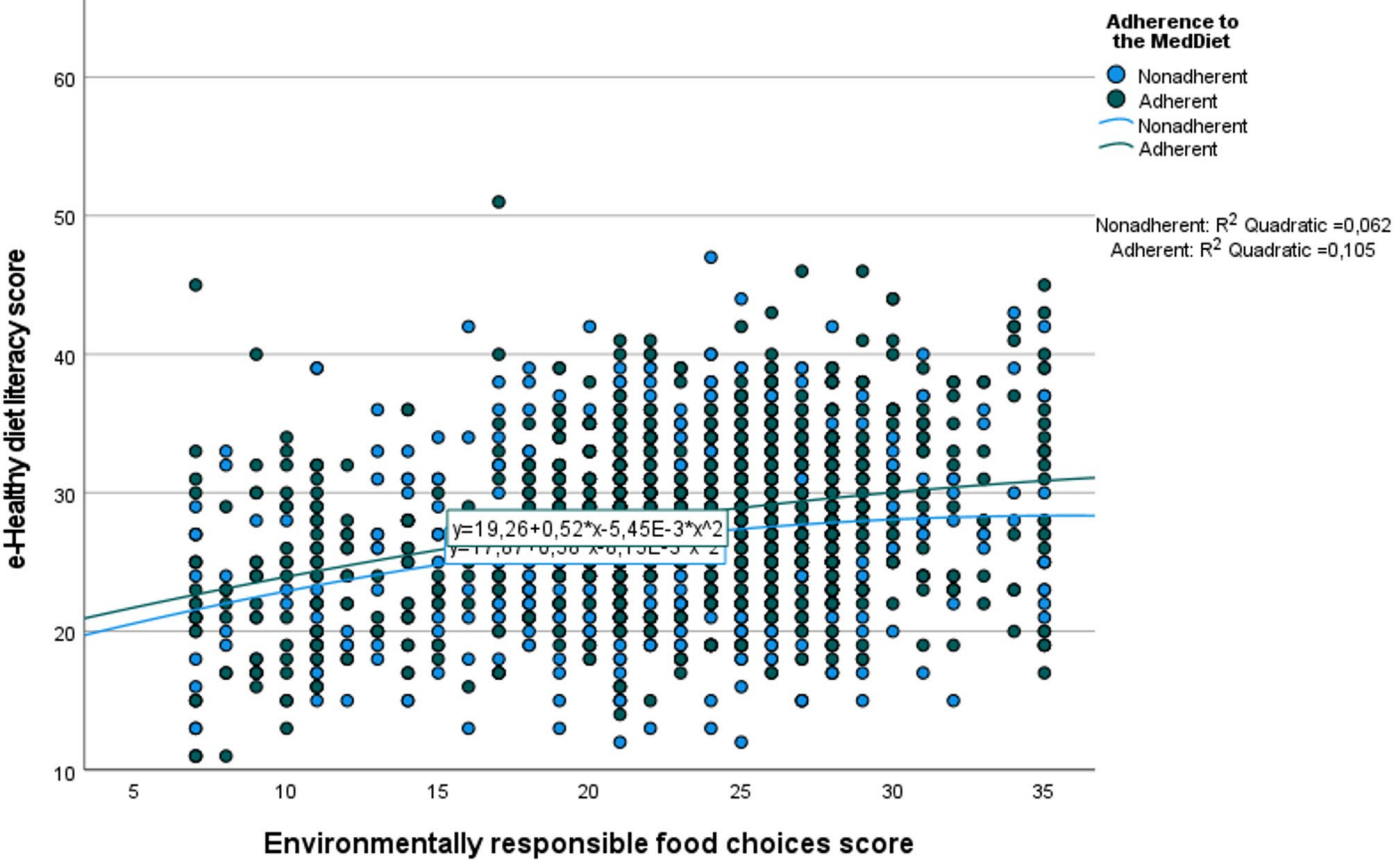
The relationship between e-healthy diet literacy and environmentally responsible food choices based on adherence to the Mediterranean diet. The blue and green lines represent the best-fit quadratic regression lines for nonadherent and adherent participants, respectively

Additionally, the regression model was stratified by adherence to the MedDiet. The relationship between e-HDL scores and environmentally responsible food choices was stronger among adherent participants. Among these, higher e-HDL scores explained 10.5% of the variance in environmentally responsible food choices (*p* < 0.001) (Fig. 1).

## Discussion

Digital healthy eating literacy has been increasingly associated with sustainable dietary practices and adherence to healthy eating patterns. This study sheds light on the potential relevance of digital healthy eating literacy to environmentally responsible food choices and adherence to the MedDiet. By addressing a notable gap in the literature, our findings contribute to the growing body of research on the relationship between digital healthy eating literacy, sustainable dietary practices and environmental health. Higher e-HDL scores were significantly associated with increased environmentally responsible food choices and greater adherence to the MedDiet. These results underscore the potential importance of digital healthy eating literacy in relation to sustainable dietary behaviours and environmental health.

The proliferation of internet-based healthy eating platforms has made digital healthy eating literacy increasingly important. While such platforms offer the advantages of reaching large audiences, cost-effectiveness, and influencing consumer behaviour, the information provided may often be biased, incomplete, misleading, or commercially motivated [12]. Nutrition literacy has been shown to be a key factor in shaping healthy or unhealthy food choices [20]. In both developed and developing countries, interest in environmentally friendly foods has significantly increased in recent years [16]. Food choices are a critical area for improving environmental sustainability, as they account for approximately one-third of a household’s total environmental impact [1]. High-impact foods such as meat, dairy products, and ultra-processed items contribute significantly to environmental degradation due to their energy-intensive production, land use, and greenhouse gas emissions. In contrast, plant-based alternatives like grains, fruits, and vegetables are key to reducing emissions and supporting environmental sustainability [2, 5, 11].

To date, no study has explicitly examined the relationship between digital healthy eating literacy and environmentally responsible food choice scores. However, previous research has linked nutrition literacy with sustainable eating behaviours [4, 5, 11]. For example, Mortaş et al. [11] reported that increased nutrition literacy is associated with behaviours such as seasonal food consumption, reduced food waste, decreased meat consumption, and healthier dietary patterns. Similarly, Lee et al. [4] found that knowledge of interpreting food labels is associated with ecological eating behaviours, particularly among women. Our findings align with these results, showing that higher e-HDL scores are associated with stronger environmentally responsible food choices. This relationship was more pronounced among non-smokers, non-drinkers, those exercising daily, and individuals with higher income levels. These trends may be explained by the likelihood that health-conscious individuals are more informed about their food choices, while higher-income individuals can afford environmentally friendly foods, which are often more expensive.

In addition to food choices, the sustainability of the diet itself is crucial for environmental health. The MedDiet, with its emphasis on plant-based foods and limited consumption of animal-based products, offers dual benefits for personal health and environmental sustainability, making it a model dietary pattern for promoting planetary health [6]. Although no prior research has directly examined the relationship between digital healthy eating literacy and adherence to the MedDiet, studies have shown positive associations between general nutrition literacy and adherence to this dietary pattern [6, 10, 20]. For example, Taylor et al. [20] reported that individuals with lower nutrition literacy consumed more foods associated with the Western diet, while those with higher nutrition literacy preferred foods aligned with the MedDiet. In our study, higher e-HDL scores were associated with greater adherence to the MedDiet, particularly among women, non-smokers, non-drinkers, and those with lower or equal income levels. For instance, women and non-smokers may exhibit greater health awareness [21, 22], which may help explain stronger adherence to sustainable dietary practices such as the MedDiet. Additionally, the higher cost of animal-based foods limited in the MedDiet may make this pattern more accessible to individuals with lower or equal income levels, who naturally consume more plant-based alternatives. Interestingly, adherence to the MedDiet al.so appeared to strengthen the association between e-HDL scores and environmentally responsible food choices, suggesting a potential reinforcing relationship between dietary patterns and digital healthy eating literacy. This interaction underscores the potential for integrating sustainable diets and targeted nutrition education to enhance environmentally conscious behaviours.

### Practical implications

These findings have practical implications for public health nutrition, particularly in an era where digital platforms play an increasingly influential role in disseminating dietary information. Given the widespread availability of unregulated and often conflicting nutrition content online, individuals must not only access information but also develop the ability to critically evaluate its accuracy and credibility. Digital healthy eating literacy may serve as a foundational skill that helps consumers navigate this landscape, identify reliable sources, and make informed decisions aligned with sustainable and health-promoting dietary behaviours. The e-HDL questionnaire used in this study may also serve as a useful screening tool for dietitians and healthcare professionals to assess literacy levels and inform the design of tailored nutrition education. Existing literature suggests that factors such as the credibility of the account owner (e.g. health professionals vs. influencers), content format (e.g. video, infographic, text), and posting frequency may influence how users perceive, trust, and act upon nutrition information [23, 24]. Although these dimensions were not directly measured in this study, they warrant further investigation in future research. Public health interventions could benefit from incorporating digital nutrition literacy and sustainability-related content into their strategies, including public campaigns, school-based curricula, and community-level programmes, to encourage individual behaviour change and environmental sustainability. In this digital age, evaluating individuals’ ability to interpret, question, and apply nutrition information is essential to improving both dietary practices and environmental outcomes.

### Strengths, limitations, and future research

This study has several strengths. It is among the first to examine the association between digital healthy eating literacy, environmentally responsible food choices and adherence to the MedDiet. Additionally, the use of a regression model stratified by adherence to the MedDiet provides a novel perspective on these relationships. The large sample size and inclusion of participants from all geographic regions of Türkiye further enhance the generalisability of the findings.

However, the study has some limitations. First, anthropometric measurements were self-reported, which may have introduced bias. Second, the online survey method may have led to the inclusion of younger and more educated participants, potentially limiting the generalisability to other demographic groups. This limitation may reflect the greater ease with which younger, educated individuals engage in online surveys. Third, the sample may have been biased toward individuals who were already health-conscious, as participants were required to follow at least one social media account related to healthy eating. This pre-existing interest may have inflated e-HDL scores and further limited generalisability. Fourth, the study did not collect data on the specific characteristics or reputability of the social media accounts followed, which may have influenced participants’ levels of trust, engagement, and digital literacy. Last, dietary intake was not directly assessed; instead, environmentally responsible food preferences and the MedDiet adherence were measured using validated self-report scales.

Future studies could benefit from incorporating objective dietary intake data, examining the characteristics and reputability of social media sources, and employing more diverse sampling strategies to validate and expand upon these findings.

## Conclusion

The findings suggest that digital healthy eating literacy is positively associated with environmentally responsible food choices and adherence to the MedDiet. Raising awareness about the accuracy and reliability of nutrition-related information on digital platforms and developing appropriate educational content could contribute to sustainable eating behaviours and environmental health. Future policies should focus on enhancing digital healthy eating literacy to help support sustainable diets and planetary health.

## Electronic supplementary material

Below is the link to the electronic supplementary material.

Supplementary Material 1

## Data Availability

The datasets used and/or analysed during the current study are available from the corresponding author on reasonable request.

## Abbreviations

BMI: Body mass index
CI: Confidence intervals
e-HDL: e-healthy diet literacy
MEDAS: Mediterranean diet adherence screener
MedDiet: Mediterranean diet
OR: Odds ratio

## References

[1] Eldesouky A, Mesias FJ, Escribano M. Perception of spanish consumers towards environmentally friendly labelling in food. Int J Consumer Stud. 2020;44(1):64–76. 10.1111/ijcs.12546.

[2] Hartmann C, Lazzarini G, Funk A, Siegrist M. Measuring consumers’ knowledge of the environmental impact of foods. Appetite. 2021;167:105622. 10.1016/j.appet.2021.105622. 34363900 10.1016/j.appet.2021.105622

[3] Alcorta A, Porta A, Tárrega A, Alvarez MD, Vaquero MP. Foods for plant-based diets: challenges and innovations. Foods. 2021;10(2):293. 10.3390/foods10020293. 33535684 10.3390/foods10020293 PMC7912826

[4] Lee Y, Kim T, Jung H. Effects of university students’ perceived food literacy on ecological eating behavior towards sustainability. Sustainability. 2022;14(9):5242.

[5] Kabasakal-Cetin A, Aksaray B, Sen G. The role of food literacy and sustainable and healthy eating behaviors in ultra-processed foods consumption of undergraduate students. Food Qual Prefer. 2024;119:105232. 10.3390/su14095242.

[6] Aureli V, Rossi L. Nutrition knowledge as a driver of adherence to the mediterranean diet in italy. Front Nutr. 2022;9:804865. 10.3389/fnut.2022.804865. 35387192 10.3389/fnut.2022.804865 PMC8978558

[7] Kocaadam-Bozkurt B, Bozkurt O. Relationship between adherence to the mediterranean diet, sustainable and healthy eating behaviors, and awareness of reducing the ecological footprint. Int J Environ Health Res. 2023;33(4):430–40. 10.1080/09603123.2023.2172384. 36726049 10.1080/09603123.2023.2172384

[8] Fresán U, Martínez-Gonzalez M-A, Sabaté J, Bes-Rastrollo M. The mediterranean diet, an environmentally friendly option: evidence from the seguimiento universidad de navarra (SUN) cohort. Public Health Nutr. 2018;21(8):1573–82. 10.1017/S1368980017003986. 29380717 10.1017/S1368980017003986 PMC10261578

[9] Grosso G, Fresán U, Bes-Rastrollo M, Marventano S, Galvano F. Environmental impact of dietary choices: role of the mediterranean and other dietary patterns in an italian cohort. Int J Environ Res Public Health. 2020;17(5):1468. 10.3390/ijerph17051468. 32106472 10.3390/ijerph17051468 PMC7084186

[10] Depboylu GY, Kaner G, Süer M, Kanyılmaz M, Alpan D. Nutrition literacy status and its association with adherence to the mediterranean diet, anthropometric parameters and lifestyle behaviours among early adolescents. Public Health Nutr. 2023;26(10):2108–17. 10.1017/S1368980023001830. 37622233 10.1017/S1368980023001830 PMC10564606

[11] Mortaş H, Navruz-Varlı S, Çıtar-Dazıroğlu ME, Bilici S. Can unveiling the relationship between nutritional literacy and sustainable eating behaviors survive our future? Sustainability. 2023;15(18):13925. 10.3390/su151813925.

[12] Van Duong T, Chiu C-H, Lin C-Y, Chen Y-C, Wong T-C, Chang PW, et al. E-healthy diet literacy scale and its relationship with behaviors and health outcomes in taiwan. Health Promot Int. 2021;36(1):20–33. 10.1093/heapro/daaa033. 32267935 10.1093/heapro/daaa033

[13] Silk KJ, Sherry J, Winn B, Keesecker N, Horodynski MA, Sayir A. Increasing nutrition literacy: testing the effectiveness of print, web site, and game modalities. J Nutr Educ Behav. 2008;40(1):3–10. 10.1016/j.jneb.2007.08.012. 18174098 10.1016/j.jneb.2007.08.012

[14] World Health Organization. Obesity: preventing and managing the global epidemic: report of a WHO consultation. 2000. https://apps.who.int/iris/handle/10665/42330. Accessed 1 Dec 2024. 11234459

[15] Karahan Yılmaz S, Eskici G, Sarac OE. Validity-reliability of the e-Healthy diet literacy scale in turkish adults. Baltic J Health Phys Activity. 2023;15(3):9. 10.29359/BJHPA.15.3.09.

[16] Başar Ş, Başar EE. How does the environmental knowledge of turkish households affect their environmentally responsible food choices? The mediating effects of environmental concerns. Int J Agric Environ Food Sci. 2020;4(3):348–55. 10.31015/jaefs.2020.3.14.

[17] Martínez-González MÁ, Corella D, Salas-Salvadó J, Ros E, Covas MI, Fiol M, et al. Cohort profile: design and methods of the PREDIMED study. Int J Epidemiol. 2012;41(2):377–85. 10.1093/ije/dyy225. 21172932 10.1093/ije/dyq250

[18] Pehlivanoğlu EFÖ, Balcıoğlu H, Ünlüoğlu İ. Turkish validation and reliability of mediterranean diet adherence screener. Osmangazi J Med. 2020;42(2):160–4. 10.20515/otd.504188.

[19] Turkish Statistical Institute. World population day (In Turkish). 2024. https://data.tuik.gov.tr/Bulten/Index?p=World-Population-Day-2024-53680&dil=2. Accessed 1 Dec 2024.

[20] Taylor MK, Sullivan DK, Ellerbeck EF, Gajewski BJ, Gibbs HD. Nutrition literacy predicts adherence to healthy/unhealthy diet patterns in adults with a nutrition-related chronic condition. Public Health Nutr. 2019;22(12):2157–69. 10.1017/S1368980019001289. 31146797 10.1017/S1368980019001289 PMC6827561

[21] Lohse T, Rohrmann S, Bopp M, Faeh D. Heavy smoking is more strongly associated with general unhealthy lifestyle than obesity and underweight. PLoS ONE. 2016;11(2):e0148563. 10.1371/journal.pone.0148563. 26910775 10.1371/journal.pone.0148563 PMC4765891

[22] Ek S. Gender differences in health information behaviour: a finnish population-based survey. Health Promot Int. 2015;30(3):736–45. 10.1093/heapro/dat063. 23985248 10.1093/heapro/dat063

[23] Kreft M, Smith B, Hopwood D, Blaauw R. The use of social media as a source of nutrition information. South Afr J Clin Nutr. 2023;36(4):162–8. 10.1080/16070658.2023.2175518.

[24] Chan V, Allman-Farinelli M. Young australian adults prefer video posts for dissemination of nutritional information over the social media platform instagram: A pilot cross-sectional survey. Nutrients. 2022;14(20):4382. 10.3390/nu14204382. 36297066 10.3390/nu14204382 PMC9610946

