# Supplementary material for: Digital healthy eating literacy: its role in sustainable food choices and mediterranean diet adherence
Source: BMC Public Health. 2025 Jun 5;25:2109. doi: 10.1186/s12889-025-23353-4 (PMC12142959; doi:10.1186/s12889-025-23353-4)
Supplement: Supplementary file 1 — Supplementary Material 1 [file 12889_2025_23353_MOESM1_ESM.docx]

**Supplementary Table 1.** E-Healthy Diet Literacy Questionnaire

***In the last year, how often have you…***

| ***Finding domain*** | **Never** | **A few times a year** | **A few times a month** | **A few times a week** | **Daily** |
| --- | --- | --- | --- | --- | --- |
| …searched for healthy diet information from Internet during last 1 year? |  |  |  |  |  |
| …searched for healthy diet information from the Institutional/official channels: Public research institutes, governmental institutions, ministry of health, health promotion administration, hospital website, . . .? |  |  |  |  |  |
| …searched for healthy diet information from the Non-institutional channels: Advertisement, commercial page, blog, . . .? |  |  |  |  |  |

***Is it correct or wrong for the below information as it often appeared on the Internet?***

| ***Understanding domain*** | **Yes** | **No** | **I don’t know** |
| --- | --- | --- | --- |
| ...do not eat starch can achieve weight loss effect? |  |  |  |
| ...to control blood sugar, should try to avoid eating sweet fruit? |  |  |  |
| ...if you worry about high cholesterol, should try to avoid eating egg yolk? |  |  |  |
| ...‘food additives are harmful to the human body’? |  |  |  |

***To what extent could you say…?***

| ***Judging domain*** | **Strongly disagree** | **Disagree** | **Unsure** | **Agree** | **Strongly agree** |
| --- | --- | --- | --- | --- | --- |
| ...the online healthy diet information provided from institutional channels is more trustworthy than those from non-institutional channels? |  |  |  |  |  |
| ...the online healthy diet information provided by dietitians, healthcare providers are usually more trustworthy than other sources? |  |  |  |  |  |

***How often have you…***

| ***Applying domain*** | **Never** | **Rarely** | **Sometimes** | **Often** | **All the time** |
| --- | --- | --- | --- | --- | --- |
| … posted/commented on the incorrect online healthy diet information? |  |  |  |  |  |
| … discussed with your healthcare professional about the online healthy diet information found to  make decision? |  |  |  |  |  |

The Finding domain was measured using a 5-Likert type scale anchored by 1 (never) to 5 (daily); the Judging domain was measured using a 5-Likert type scale anchored by 1 (strongly disagree) to 5 (strongly agree); the Applying domain was evaluated using a 5-Likert type scale anchored by 1 (never) to 5 (all the time). The Understanding domain was evaluated through the options of yes/no/I don’t know. The option “yes” was worth 5 points, whereas “no” or “I don’t know” was worth 1 point. The total score is calculated by summing the scores of all items, with higher scores indicating greater e-HDL. The maximum possible score is 55, representing the highest level of e-HDL.

**Supplementary Table 2.** Environmentally Responsible Food Choice Scale

|  | **Strongly disagree** | **Disagree** | **Unsure** | **Agree** | **Strongly agree** |
| --- | --- | --- | --- | --- | --- |
| I can pay more for organically grown food |  |  |  |  |  |
| I avoid consuming food with GMO (genetically modified organism) |  |  |  |  |  |
| I prefer to consume eco-label food |  |  |  |  |  |
| I am careful not to consume too much meat |  |  |  |  |  |
| I prefer to buy dairy products from local producers |  |  |  |  |  |
| I avoid consuming imported food such as a variety of exotic fruits |  |  |  |  |  |
| I avoid consuming canned “ready-made” food |  |  |  |  |  |

This seven-item scale uses a five-point Likert scale, ranging from 1 (strongly disagree) to 5 (strongly agree). The total score is calculated by summing the scores of all items. Higher scores indicate a stronger preference for environmentally responsible food consumption. The maximum score is 35.

**Supplementary Table 3.** Mediterranean Diet Adherence Screener

|  | Do you use olive oil as the principal source of fat for cooking? | ( ) At least 2 times a week  ( ) Less than 2 times a week |
| --- | --- | --- |
|  | How much olive oil do you consume per day (including that used in frying, salads, meals eaten away from home, etc.)? (1 tablespoon=13.5 g) | ( ) More than 48 grams (3.5 tablespoons)  ( ) Less than 48 grams (3.5 tablespoons) |
|  | How many servings of vegetables do you consume per day? Count garnish and side servings as 1/2 point; a full serving is 200 g. | ( ) 2 servings or more per day  ( ) Less than 2 servings per day |
|  | How many pieces of fruit (including fresh-squeezed juice) do you consume per day? | ( ) 3 servings or more per day  ( ) Less than 3 servings per day |
|  | How many servings of red meat, hamburger, or sausages do you consume per day? A full serving is 100–150 g. | ( ) 100 grams per day and more  ( ) Less than 100 grams per day |
|  | How many servings of butter, margarine, or cream do you consume per day? (1 tablespoon= 12 g) | ( ) 1 tablespoon or more per day  ( ) Less than 1 tablespoon per day |
|  | How many carbonated and/or sugar-sweetened beverages do you consume per day? (1 portion= 100 ml) | ( ) 1 portions per day and more  ( ) Less than 1 portions per day |
|  | Do you drink wine? How much do you consume per week? | ( ) No, I don't consume  ( ) Yes, 7 cup or more per week  ( ) Yes, less than 7 cup per week |
|  | How many servings of pulses do you consume per week? (1 portion= 150 g) | ( ) 3 servings or more per week  ( ) Less than 3 servings per week |
|  | How many servings of fish/seafood do you consume per week? (1 portion = 100-150 g of fish or 200 g of seafood) | ( ) 3 servings or more per week  ( ) Less than 3 servings per week |
|  | How many times do you consume commercial (not homemade) pastry such as cookies or cake per week? | ( ) 3 servings or more per week  ( ) Less than 3 servings per week |
|  | How many times do you consume nuts per week? ( (1 portion = 30 g) | ( ) 3 servings or more per week  ( ) Less than 3 servings per week |
|  | Do you prefer to eat chicken, turkey or rabbit instead of beef, pork, hamburgers, or sausages? | ( ) Yes  ( ) No |
|  | How many times per week do you consume boiled vegetables, pasta, rice, or other dishes with a sauce of tomato, garlic, onion, or leeks sauted in olive oil? | ( ) 2 or more times a week  ( ) Less than 2 times a week |

One point was given for using olive oil as the principal source of fat for cooking, preferring white meat over red meat, or for consuming:

1) More than 48 grams (3.5 tablespoons) (1 tablespoon = 13.5 g) of olive oil/d (including that used in frying, salads, meals eaten away from home, etc.).

2) 2 or more servings of vegetables/day.

3) 3 or more pieces of fruit/day.

4) Less than 100 grams of red meat or sausages/day.

5)Less than 1 tablespoon of animal fat/day.

6) Less than 1 portions of sugar-sweetened beverages/day.

7) 7 or more cup of red wine/week.

8) 3 or more servings of pulses/week.

9) 3 or more servings of fish/week.

10) fewer than 3 commercial pastries/week.

11) 3 or more servings of nuts/week or

12) 2 or more servings/week of a dish with a traditional sauce of tomatoes, garlic, onion, or leeks saute´ed in olive oil. If the condition was not met, 0 points were recorded for the category.

With total scores of 7 or higher indicating acceptable adherence and scores of 9 or higher reflecting strict adherence to the Mediterranean Diet, the final PREDIMED score ranged from 0 to 14.
